# Seroprevalence of dengue virus among children presenting with febrile illness in some public health facilities in Cameroon

**DOI:** 10.11604/pamj.2018.31.177.16390

**Published:** 2018-11-13

**Authors:** Salomon Bonsi Tchuandom, Thibau Flaurant Tchouangueu, Christophe Antonio-Nkondjio, Abel Lissom, Jean Olivier Ngono Djang, Etienne Philemon Atabonkeng, Assumpta Kechia, Godwin Nchinda, Jules-Roger Kuiate

**Affiliations:** 1 Department of Biochemistry, University of Dschang, Cameroon; 2 Public School of medical Laboratory Technicians, Yaoundé, Cameroon; 3 Laboratory of Microbiology and Immunology, Chantal Biya International Reference Centre for Research and Prevention on HIV/AIDS Yaoundé; 4 Laboratoire de Recherche sur le Paludisme, Organisation de Coordination pour la lutte Contre les Endémies en Afrique Centrale (OCEAC), Yaoundé, Cameroun; 5 Department of Animal Biology and Physiology, University of Yaoundé 1, Yaoundé; 6 Ministry of Public Health, Yaoundé, Cameroun

**Keywords:** Dengue virus, children, febrile illness, health facilities, Cameroon

## Abstract

**Introduction:**

A routine diagnosis of Dengue virus (DENV) infection is not usually conducted in hospitals. Because symptoms overlap, many potential febrile illnesses due to DENV may be confused for malaria, typhoid or paratyphoid (enteric) fever. The absence of data on DENV exposure rates among children attending health facilities could undermine management of this disease. This study aimed to investigate the seroprevalence of dengue virus infection in children presenting febrile illness in some public health facilities in Cameroon.

**Methods:**

A cross-sectional study was performed in children ≤ 15 years attending seven urban and three semi-urban public hospitals of Cameroon. From each volunteer, 2ml of whole blood was collected and tested for dengue virus IgM, malaria (Pf/Pan antigens) and enteric fever (Typhoid IgM) using rapid diagnostic tests (RDT); in order to allow the healthcare workers to quickly put the positive cases under appropriate treatment. Positive cases of dengue virus infection were confirmed by indirect ELISA. Data analysis were performed using the statistical package for social sciences software, version 22.1.

**Results:**

A total of 961 children were enrolled in the study and 492 (51.2%) were infected with at least one of the three pathogens. Overall, DENV IgM seroprevalence among febrile children was 14.4% (138/961). About 390 (40.6%) and 22 (2.3%) had malaria (Pf/Pan Ag) and enteric fever (Typhoid IgM) respectively. Co-infection with dengue virus was found in 51 (5.3%) participants. The dengue virus IgM seroprevalence was higher in Bankim (19.3%), Ntui (18.3%) and Douala (18.2%).

**Conclusion:**

Dengue virus infection seroprevalence appears to be low in children presenting with febrile illness in the studied health centres in Cameroon but call for more attention and research to further characterise the circulating strains of the dengue virus.

## Introduction

Dengue is a tropical disease transmitted by mosquitoes of the genus *Aedes* [1, 2]. Infection by dengue virus causes flu-like illness and can occasionally develop to severe complications and death. Dengue has grown dramatically over the years and it is estimated that about half of the world population is now at risk [3]. Dengue is a leading cause of serious illness and death among children in Asia and Latin America [3]. Yet the real number of dengue cases is underreported and many cases are misclassified [3]. In Africa, at least 15 countries declared locally acquired dengue cases since 1960. Moreover, dengue has frequently been detected in travellers returning from over 30 African countries. In Central African region, dengue is highly prevalent and cases of dengue infection have been reported in recent dengue-like symptoms outbreaks in Cameroon and Gabon [4-6]. A study conducted in three major towns in Cameroon by Demanou *et al.* [6], reported dengue virus IgM seroprevalence of 0.3, 0.1 and 0.0% among healthy children in Douala, Garoua and Yaounde respectively but no known study has described DENV IgM seroprevalence among febrile children in Cameroon. WHO gives priority to malaria when considering the aetiology of fever in tropical countries [7].

It is therefore recommended to think of malaria diagnosis in the first place when feverish syndromes are observed in patients with empirical treatment of malaria administered in sufficient dose and duration. Like malaria or enteric fever, dengue infection symptoms include fever headache, rash, vomit and joint pain [8, 9]. Fever accounts for 70% of purposes of consultation for children visiting health care facilities in Cameroon [10]. More than 80% of primary infection of dengue virus in children are usually asymptomatic and are generally characterized by a fever greater than 38°C in addition to other symptoms identical to those of malaria or enteric fever [11, 12]. A particular feature for dengue fever is that its re-infections can be associated with haemorrhage or shock syndromes in patients [2, 6, 12-19]. Children under the age of 15 are particularly at risk of developing a severe form of dengue fever (DF) as well as any patient who had already been infected by another serotype of the virus [20]. Most arboviruses especially DENV are rarely taken into consideration by local clinicians because the disease is not considered as endemic and the diagnosis is always focused on other endemic diseases such as enteric fever and malaria. However, early diagnosis of DF by a rapid diagnostic test for the detection of immunoglobulin M (IgM) and non-structural protein 1 (NS1) is important for preventing potential complications in children [21, 22] and also to limit the over-consumption of anti-malarial drugs and antibiotics by patients who do not need them. This could also help in slowing down the development and emergence of resistance to antibiotics and antimalarial drugs.

## Methods

**Study design**: this study was carried out in 10 public health facilities located in the Adamaoua, Center, Far North, Littoral and West regions of Cameroon. These health care centres are tertiary health facilities serving the low, middle and high income patients. The official languages spoken are French and English.

**Research design**: this was a cross-sectional multicentric study performed to determine the seroprevalence of dengue virus infection among children presenting with undifferentiated fever in some public health facilities in Cameroon. The study was conducted from March 2016 to April 2017. In absence of data on DENV exposure rates among children attending health facilities with febrile illness in Cameroon, maximum possible proportion was considered to be 0.5 based on [23] and the required sample size calculated to be 384. The study included 961 children aged 0-15 years randomly selected from health facilities. Systematic random sampling was employed by selecting after every third child. Children included in this study were febrile (temperature ≥38°C) with at least one of specific symptoms (fever, headache, rash, vomit and joint pain), aged 4 months to 15 years regardless of gender, ethnicity or tribe. However, we excluded children admitted at the hospital for known diseases or with fever more than seven days.

**Figure 1:**
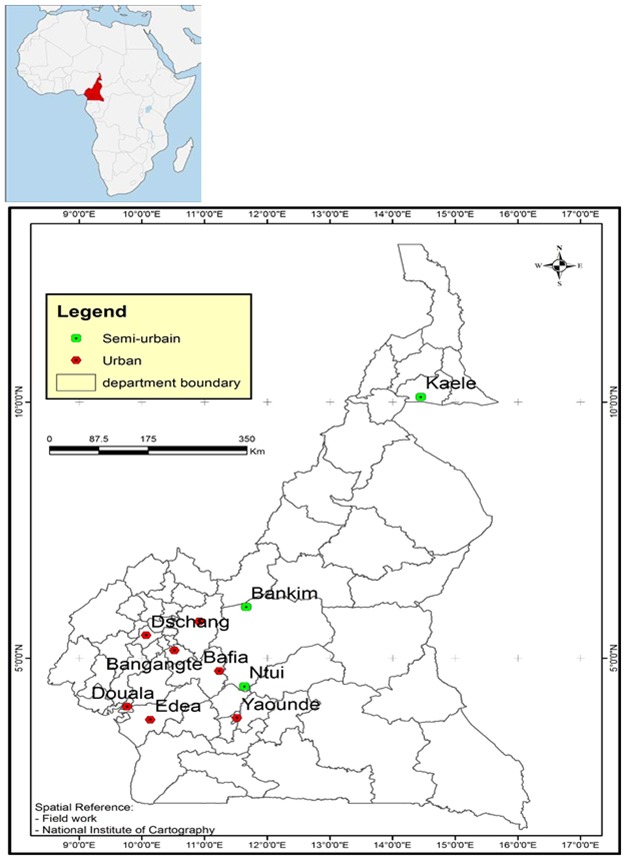
Map of Cameroon: location of the 10 study sites

**Description of study sites**: the study was conducted in ten areas (five semi-urban and five urban) including Kaelé, Bankim, Ntui, Bafia, Edéa, Yaoundé, Douala, Bangangte, Foumban, Dschang. The populace are predominantly traders, house wives and students. The map was constructed using the software ArcGIS^®^ version 10.2 and the field coordinates were obtained with the help of GPS etrex 20X (Garmin^®^) as shown on Figure 1. Kaelé (10° 06' 00"N, 14° 27' 00" E): is a semi-urban city located in a dry savanna or sudano-sahelian zone characterized by a long dry season of about eight months, (October to May) and a short rainy season of four months (June to September). The average annual rainfall and temperature are 809mm 28.1°C respectively. The health district of Kaelé covers a population of 126,376 inhabitants from 90 villages.

Bankim (6° 00' 00" N, 11o 40' 00" E): is a semi-urban city located in a wet savanna zone characterized by 5 months of dry season and 7 months of rains with an annual rainfall of 1200mm. The health district of Bankim cover 43 villages for a total population of 50,000 inhabitants. Bangangte (5° 09'00"N, 10° 31' 00" E), Foumban (5° 43' 00" N, 10° 55' 00" E) and Dschang (5° 23' 00" N, 10° 10" 00" E): are urban cities all located in the high mountain area of western Cameroon. The area exhibits, a sub-tropical climate characterized by two seasons; one dry season of 4 months and one rainy season of 8 months. Annual average rainfall and temperature are of 321 mm and 21.6°C respectively. The health district of Bangangte covers 91 villages for a population of 63,595 inhabitants, Foumban health district covers 15 villages for a total of 152,728 inhabitants and Dschang health district covers 11 villages for a total of 101,385 inhabitants.

Ntui (4° 26' 59.99" N, 11° 37' 59.99" E), Bafia (4° 45' 00" N, 11° 14' 00" E), Yaoundé (03° 50' 00" N, 11° 31' 00" E) and Edéa (03° 48' 00" N, 10° 08' 00" E) are all located in the equatorial forest zone characterized by four seasons including 2 rainy seasons and 2 dry seasons. The climate is equato-Guinean (with average annual rainfall ranging from 1200 to 3300mm). Ntui is a semi-urban city whereas Bafia, Yaoundé and Edéa are all urban areas. The health district of Ntui covers 27 villages for a total population of 20, 000 inhabitants, Bafia cover 8 villages for a total population of 55,506 inhabitants and Yaoundé the capital of Cameroon has a population of 2,765,568 inhabitants with many reference health centres [24]. Douala (04° 03' 8.1" N, 09° 45' 42.94" E): is an urban area located in the coastal zone characterized by two seasons (01 dry (03 months), 01 humid (9 months of rain). The climate is monsoon humid (average annual precipitation and temperature of 4000 mm of rain per year and 28°C respectively). Douala is the economic capital and has a population of 2,768,436 inhabitants with many reference health care centres [24].

**Ethical consideration**: this study received ethical approval from the Cameroonian National Ethics Committee for Research on Human Subjects, Ethical Clearance number N° 2014/12/530/CE/CNERSH/SP in accordance with the Helsinki declaration by the World Medical Association (WMA) on the ethical principles for medical research involving human subjects [25]; after which the subject were served questionnaires.

**Patient information and informed consent**: the risks, if any and benefits of the study participation were explained to the patient during the administration of informed consent. Written consent was obtained from the parents or guardians or legal representative. In case of patients between 10-14 years, assent was also obtained.

**Blood sampling**: two millilitres (2ml) of venous blood was collected by venipuncture from children and dispensed in EDTA tubes and centrifuged at 4000 rpm for 10 min for rapid diagnostic test of enteric fever, malaria, dengue markers (IgM) and IgM ELISA assay. The plasma fraction was collected, aliquoted in small, single used volumes and stored for at -20° for subsequent analyses.

**Detection of enteric fever**: The diagnosis of enteric fever was done using SD Bioline Typhoid (Standard Diagnostics, Kyyonggi-do, Korea) method to qualitatively detect IgM and IgG antibodies to serotype Typhi antigens (unspecified). The presence of specific antibodies was revealed by the appearance of red line in the test zone (T) and control zone (C) of the assay device (Figure 1).

**Detection of plasmodium spp for malaria infection**: rapid malaria antigen test (SD BIOLINE Pf/Pan test, Biostandard Diagnostic LTD, Gurgaon, India) was done using qualitatively differential method for detecting parasite dehydrogenase (pLDH) pan specific to Plasmodium spp (*P. vivax, Plasmodium ovale and Plasmodium malariae*) and histidine rich protein II (HRP-II) specific to *Plasmodium falciparum*. The presence of *Plasmodium spp* was revealed by the appearance of red line in the test zone (T) and control zone (C) of the assay device.

**Detection of dengue virus (dengue fever)**: infection to DENV was assessed using a rapid diagnostic test detecting IgM antibodies in the plasma of participants using *tell me fast^®^* Dengue IgG/IgM antibody Combo test (Biocan Diagnostics Inc. Canada). Interpretation of the results was done in conformity to the manufacturer's instructions and the presence of DENV markers was revealed by the appearance of red line in the test zone (T) and control zone (C) of the assay device.

**DENV characterization using ELISA**: DENV infection was confirmed in participants using an in-house indirect ELISA (Unpublished). To characterize the specific DENV serotypes, a quantity of 50 ng/well of each of rDengue antigen D1,D2, D3 and D4 envelopes (recombinant protein in *E. coli*, purchase from ImmunoDX, LLC, Woburn, USA), were added to high binding 96-well flat bottom microsorp (Thermo Fisher Scientific) ELISA plates and incubated overnight at 4^o^C. The following day, plates were washed 3x with PBST (PBS with 0.05 % Tween-20) and blocked either with 3% BSA or 1x Roti block (Carl ROTH, Karlsruhe, Germany) for one hour at 37°C. Plasma samples diluted at 1/200 in 2% BSA containing-PBS, then 100 µl of the diluted antibodies were added per well of the blocked ELISA plates and incubated for two hours at 37°C. Unbound antibody was removed by washing the plates 5x (198 µl/well) with PBST. Horseradish Peroxidase (HRP) coated mouse anti-human IgG Fc (Clone JDC-10) and mouse anti-human IgM (Clone SA-DA4) antibodies (Southern Biotech, Birmingham, USA) diluted 1:4000 in 0.1x roti block (100 µl/well) were introduced. Bound conjugate was detected using ABTS One Component Microwell Substrate (Southern Biotech) and the HRP reaction stopped by adding 50 µl of a stop (Sulfuric acid, 4N) solution according to the manufacturer's protocol (Southern Biotech). The optical density was measured at 405 nm using a multiskan FC microplate reader (Thermo Fisher Scientific, USA). The sample diluent was used as a negative control. The threshold of detection was calculated as the highest OD of background + 2.5 SD of background ODs. Samples with ODs greater than the threshold were considered positive.

**Statistical analysis**: tabulation, management, and analysis of raw data were carried out using Microsoft Excel (Microsoft Inc., Redmond, WA). Statistical analysis was performed with statistical package for social sciences (SPSS) software (version 22.1). The mean, frequencies and percentages were used to summarize descriptive statistics of the data. Chi-square (x^2^) test was used to assess relationships between selected and/or categorical variables namely gender, sex, semi-urban and urban areas. The binomial tests were used to determine the statistical discrepancy of dengue virus positive children in malaria positive and malaria negative. Odds ratios were determined from contingency tables using the Fisher's exact test to assess the level of association between dengue exposure cases and dependent variable such as age, sex or sites characteristics. The level of significance will be set at p<0.05.

## Results

**Characteristics of study populations**: a total of 961 consenting children attending hospital for febrile illness and for whom the clinician suspected malaria and/or typhoid fever were included in the study. The minimum number of children recruited per site was 60 in Bafia and the highest was 150 in both Kaele and Bankim (Table 1). According to gender there were more males (51 .5%) compared with females (48.5%), with a general age mean (±SD) 7.1±2.9 years [4 months-15 years]. Majority (48.8%) of the children were between the ages of 1-5 years, followed by those aged 5-15 years (43.8%). Those age 4-11 months had the least percentage (7.4%) (Table 1).

**Table 1:** Demographic characteristics of the children with febrile illness according to sites characteristics

		Gender		Age groups				
Sites characteristics	Sites	Female	Male	< 1 yr	1-5 yrs	>5yrs	TOTAL	%
**Semi-urban**	Kaele	81	69	10	21	119	150	15.6
	Bankim	63	87	18	37	95	150	15.6
	Ntui	41	30	5	42	24	71	7.4
**Urban**	Bafia	27	33	4	36	20	60	6.2
	Edéa	37	31	3	38	27	68	7.1
	Yaoundé	59	29	13	50	22	85	8.8
	Douala	53	24	8	43	26	77	8.0
	Bangangte	54	42	3	67	26	96	9.9
	Foumban	29	72	2	83	16	101	10.5
	Dschang	22	81	5	52	46	103	10.7
**TOTAL**		466	495	71	469	421	**961**	**100%**

yr (s): year(s)

**Prevalence of different infections**: out of the 961 children examined, 138 (14.36%) were positive for dengue virus IgM and 390 (40.6%) had evidence of malaria parasite infection. *Plasmodium falciparum* was the most frequent species identified (377/390). Other *Plasmodium spp* accounted for 14.4% (13/390) of Plasmodium infections. DENV specific antibodies were detected in individuals from all study sites with Bankim, Yaoundé and Dschang, having the highest exposure rates (Table 2). A high prevalence of malaria was recorded in Douala, Edéa and Bafia whereas low infection rate was recorded in Kaelé where transmission is seasonal (Table 2). Seropositivity rates for each infection tested differ according to sites. The diagnosis of enteric fever detected 22 (2.3%) infection cases. The pathogenic agents detected could be *Salmonella typhi* or *Salmonella paratyphi* (Table 2). Out of 961 children, 40 (4.2%) and 6 (0.6%) cases of co-infections for malaria and dengue or malaria and typhoid were detected respectively. Pattern of clinical signs among febrile illness shows association between clinical signs (joint pain and fatigue) and sero-positivity to dengue (p<0.0001) (Table 3). Table 4 summarizes the co-infection of dengue virus and malaria status among febrile illness. More (18.42%) of negative malaria children were dengue virus positive, while a lower but not significant percentage (8.44%) of positive malaria children were dengue virus positive (p=0.075). No significant association was detected between seropositivity and exposure to DENV and categorical variables (age, sex, collection sites) (Table 5).

**Table 2:** Prevalence of different infections in study sites

			Mono-infections n (%)			Co-infections n (%)		
Sites	Study site	Number tested	Mal	Anti-DENV IgM	Sal	Mal + DENV	Mal + Sal	Negative
**Semi-urban**	Kaele	150	19 (12.7)	19 (12.7)	7 (4.7)	2 (5.3)	2 (1,3)	63 (42.0)
	Bankim	150	30 (20.0)	23 (15.3)	8 (5.3)	5 (3.3%)	2 (1,3)	95 (63.3)
	Ntui	71	36 (50.7)	12 (16.9)	1 (1.4)	1 (1.4)	1 (1.4)	43 (60.5)
**Urban**	Bafia	60	33 (55.0)	9 (15)	0 (0.0)	2 (3.3)	0 (0.0)	44 (73.3)
	Edea	68	38 (55.9)	12 (17.6)	0 (0.0)	6 (8.8)	0 (0.0)	27 (39.7)
	Yaoundé	85	30 (35.3)	14 (16.5)	2 (2.4)	11 (16.2)	1 (1.2)	42 (61.7)
	Douala	77	47 (61.0)	18 (23.4)	1 (1.3)	0 (0)	0 (0.0)	53 (63.8)
	Bangangte	96	47 (49.0)	10 (10.4)	0 (0.0)	7 (7.3)	0 (0.0)	28 (29.2)
	Foumban	101	53 (52.5)	9 (8.9%)	1 (1.0)	7 (6.9%)	0 (0.0)	36 (35.6)
	Dschang	103	57 (55.3)	12 (11.7)	2 (1.9)	10 (9.7)	0 (0.0)	32 (31.1)
**TOTAL**		961	390 (40.6)	138 (14.4)	22 (2.3)	51 (5.3)	06 (0.6)	469 (48.2)

**Mal:** malaria, **Sal:** Salmonella, **DENV:** dengue virus

**Table 3:** Pattern of clinical signs among febrile illness

Clinical signs	No. of participants presenting clinical signs	No. of participants dengue virus positive (%)		p-value
Head ache	335	27	24.18	0.583
Joint pain	665	76	14.29	< 0.0001^***^
Back ache	70	10	22.86	1.000
Vomiting	7	0	0.00	0.360
Fatigue	217	27	37.33	< 0.0001^***^

**Table 4:** Co-infection of dengue virus and malaria among febrile illness

Malaria status	No of subjects tested	No. of participants dengue virus positive (%)		p-value
Negative	570	105	18.42	0.076
Positive	391	33	8.44	

**Table 5:** Odds of seropositivity according to age, sex and sites characteristics

	No tested	%IgM	OR	OR (95% CI)	P
**Age group**					
< 1 year	71	7%	1		
1-5 years	469	16%	2.5	0.98 - 6.44	0.055
6-15 years	421	14%	2.1	0.81 - 5.45	0.120
**Sex**					
Female	466	21%	1		
Male	495	24%	1.2	0.88 - 1.64	0.210
**Sites**					
Semi-urban	371	23%	1		
Urban	590	23%	1.01	0.74 - 1.38	0.930

## Discussion

Dengue virus is an important emerging disease of the tropical and sub-tropical country today. The differential diagnosis associated with dengue fever includes a wide variety of parasitic and bacterial infections that produce similar syndrome. This study investigated the seroprevalence of dengue among children presenting with undifferentiated fever who are living in ten areas of Cameroon. Laboratory diagnosis of acute infection (dengue, enteric fever or malaria) is important to provide appropriate treatment and to reduce development and emergence of resistance to antibiotics and antimalarial drugs. In malaria endemic areas like in Cameroon, most febrile illnesses are treated as malaria or enteric fever cases due to lack of trained staff and laboratory to properly rule-out malaria or enteric fever as a cause of fever and as well, identify alternative fever-causing pathogens [14, 26, 27]. Diagnosis of dengue in children based on clinical presentation is difficult as for enteric fever or malaria even if results in this study reported associations between clinical signs with most tropical diseases. The prevalence of malaria was highest, about 41% of the children complaining for fever attending hospital were really suffering from malaria. This is consistent with the actual situation of the disease in the country [24]. *Plasmodium falciparum* was the main species detected in all malaria cases. It is rather possible that a high proportion of children complaining of fever detected as negative could be suffering from malaria since the consumption of antimalarial drugs when suffering from fever before attending health care facilities have been reported to be high in the population [28]. We report 5.3% (51/961) cases of dengue with acute malaria, concurrent infections of dengue and malaria data in Cameroon are scarce. The prevalence of enteric fever (*Salmonella typhi* and *Salmonella paratyphi* A) appeared to be low in children at the different geographical settings and is in conformity with studies reported in Cameroon by Nsutebu *et al.* [14].

We determined an overall prevalence of 14.4% (138/961) of dengue among children with febrile illness in both urban and semi-urban areas. This finding is in contrast to the study carried out by Chukwuma *et al.* [29] who investigated the seroprevalence of dengue virus among children with febrile illness in Nnewi (Nigeria) and reported a prevalence rate of 77.1%. Again, the study carried out by Onoja *et al.* [30] who explored the high rate of unrecognized dengue virus infection in parts of the rainforest region of Nigeria and found a prevalence rate of 23.3%. Dengue virus IgM seroprevalence among febrile children is scarcely documented in Cameroon. However, 14.8% prevalence among children under 6 years of age agrees with a study involving in the same age group of children in Llorin, North central and in parts of the rainforest region of Nigeria, were high prevalence was reported [30, 31]. We observed few positive cases to dengue IgM (7%, 16% and 14% respectively) among the various age group of children febrile illness (less than 1 year, 1-5 years and 6-15 years old respectively). According to WHO recommendations [32], the diagnosis of dengue virus can be confirmed when at least IgM is positive during seroconversion in paired sera. As previously reported in some studies [4, 6, 8, 33] dengue is endemic in Cameroon but this study is the first carried out among children with febrile illness. Dengue virus IgM biomarkers were detected in all age groups. This suggests that children were recently bitten by *Aedes* mosquitoes during the day. However, several authors suggested transmission of antibodies or Dengue virus from mother to child through breastfeeding [34-36].

Transmission of DENV in several semi-urban and urban regions (Figure 1) is an indication that it is widespread in urban and semi-urban populations. Human-DENV-Mosquito cycle has been reportedly found in nearly all urban and semi-urban environments throughout the tropics and subtropics [37]. Dengue in tropical and subtropical regions has increased due to uncontrolled urbanization, lack of effective and sustainable vector control programs [38]. Although the serotype(s) involved in this children is/are not known, all DENV serotypes involved in urban and semi-urban dengue cycle are transmitted by domestic and peri-domestic *Aedes aegypti*and *Aedes albopictus* [20, 38-40]. These *Aedes spp* circulates steadily in Cameroon since 1984 [1, 41-48]. The seroprevalence of the dengue virus in the hospital centers included in this study was low. However, a more representative sampling, in number and spatial distribution of health centers in the Cameroonian territory would allow a more realistic prevalence. Also, the direct diagnosis by RT-PCR could show us the circulating serotypes and strains of the dengue virus in the study environment.

## Conclusion

We have reported seroprevalence of dengue virus in fever illness children, from some health facilities in Cameroon for the first time. Our study has revealed that dengue prevalence is low in children presenting with febrile illness in Cameroon. However, getting a realistic prevalence would demand a more representative sample size (numbers and multiple health facilities). Nevertheless, with this study, it has become more evident that routine dengue testing should be done in febrile illnesses children and may be adults in the hospitals of Cameroon to avoid being surprised by an epidemic due to inadequate patient follow-up. In addition, further research to determine acute fever due to dengue infection, the circulating serotypes and strains of the virus in the study environment are warranted.

### What is known about this topic

- Dengue is endemic in Cameroon;
- Malaria and enteric fever are the main causes of feverish syndromes in patients coming in consultation in health centre.

### What this study adds

- Our study provided evidence of DENV and revealed that 14.4% of fever in children were related to this virus in Cameroon. This study also brings out the need of carrying a dengue diagnosis in routine Cameroonian health facilities since this was neglected the country.

## Competing interests

The authors declare no competing interests.
